# (4-Methyl­piperazin-1-yl)(2,3,4-tri­meth­oxy­benzyl­idene)amine

**DOI:** 10.1107/S1600536814006291

**Published:** 2014-03-29

**Authors:** Channappa N. Kavitha, Jerry P. Jasinski, Manpreet Kaur, H.S. Yathirajan

**Affiliations:** a Department of Studies in Chemistry, University of Mysore, Manasagangotri, Mysore 570 006, India; b Department of Chemistry, Keene State College, 229 Main Street, Keene, NH 03435-2001, USA

## Abstract

In the title compound, C_15_H_23_N_3_O_3_, the piperazine ring is in a slightly distorted chair conformation and is twisted from the mean plane of the benzene ring making a dihedral angle of 14.94 (6)°. The 4-meth­oxy substituent is almost co-planar with the benzene ring [C—C—O—C torsion angle = 5.4 (1)°], while the meth­oxy groups at positions 2 and 3 [C—C—O—C torsion angles of 122.6 (4) and −66.1 (4)°, respectively] are twisted away from the mean plane of the benzene ring in anti­clinical and synclinical conformations, respectively. No classical hydrogen bonds or any weak inter­molecular inter­actions are observed in the crystal structure.

## Related literature   

For a review of pharmacological and toxicological information for piperazine derivatives, see: Elliott (2011 ▶). For the anti­microbial activity of Schiff base piperazine derivatives, see: Savaliya *et al.* (2010 ▶) and for their anti­bacterial activity, see: Xu *et al.* (2012 ▶). For the anti­microbial activity of piperazine derivatives, see: Kharb *et al.* (2012 ▶). For related structures, see: Kavitha *et al.* (2013*a* ▶,*b* ▶); Guo (2007 ▶); Guo & Qiu (2007 ▶); Xu *et al.* (2009 ▶); Zhou *et al.* (2011 ▶). For puckering parameters, see Cremer & Pople (1975 ▶). For standard bond lengths, see: Allen *et al.* (1987 ▶).
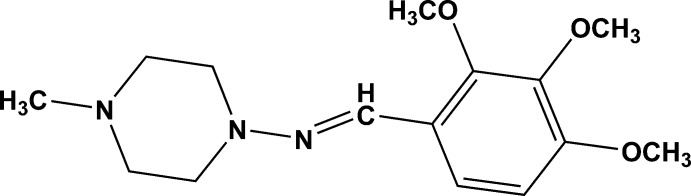


## Experimental   

### 

#### Crystal data  

C_15_H_23_N_3_O_3_
*M* *_r_* = 293.36
Orthorhombic, 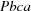

*a* = 7.84207 (14) Å
*b* = 14.2305 (3) Å
*c* = 27.6218 (5) Å
*V* = 3082.49 (10) Å^3^
*Z* = 8
Cu *K*α radiation
μ = 0.73 mm^−1^
*T* = 173 K
0.30 × 0.26 × 0.18 mm

#### Data collection  

Agilent Xcalibur (Eos, Gemini) diffractometer
Absorption correction: multi-scan (*CrysAlis PRO* and *CrysAlis RED*; Agilent, 2012 ▶) *T*_min_ = 0.290, *T*_max_ = 1.000
19693 measured reflections
2978 independent reflections
2643 reflections with *I* > 2σ(*I*)
*R*_int_ = 0.051

#### Refinement  

*R*[*F* ^2^ > 2σ(*F* ^2^)] = 0.040
*wR*(*F* ^2^) = 0.113
*S* = 1.04
2978 reflections
195 parameters
H-atom parameters constrained
Δρ_max_ = 0.23 e Å^−3^
Δρ_min_ = −0.17 e Å^−3^

### 

Data collection: *CrysAlis PRO* (Agilent, 2012 ▶); cell refinement: *CrysAlis PRO*; data reduction: *CrysAlis RED* (Agilent, 2012 ▶); program(s) used to solve structure: *SUPERFLIP* (Palatinus & Chapuis, 2007 ▶); program(s) used to refine structure: *SHELXL2012* (Sheldrick, 2008 ▶); molecular graphics: *OLEX2* (Dolomanov *et al.*, 2009 ▶); software used to prepare material for publication: *OLEX2*.

## Supplementary Material

Crystal structure: contains datablock(s) I. DOI: 10.1107/S1600536814006291/hg5389sup1.cif

Structure factors: contains datablock(s) I. DOI: 10.1107/S1600536814006291/hg5389Isup2.hkl

Supporting information file. DOI: 10.1107/S1600536814006291/hg5389Isup3.cml

CCDC reference: 992877

Additional supporting information:  crystallographic information; 3D view; checkCIF report

## Figures and Tables

**Fig. 1.**
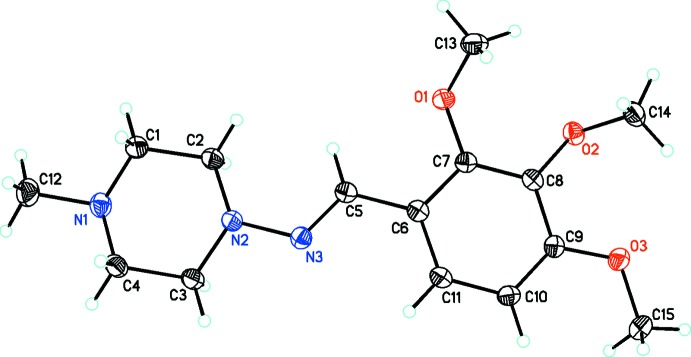
ORTEP drawing of (I) (C12H16N2O2) showing the labeling scheme with 30% probability displacement ellipsoids.

## supplementary crystallographic information

### 1. Comment

The Schiff base ligands derived from 1-amino-4-methylpiperazine have attracted the interest due to diverse biological applications found with piperazine moiety. Schiff base piperazine derivatives were found to be designed for the study of their antimicrobial activity (Savaliya *et al.*, 2010) and antibacterial activity (Xu *et al.*, 2012). A valuable insight into recent advances on antimicrobial activity of piperazine derivatives is reported (Kharb *et al.*, 2012). A review on the current pharmacological and toxicological information for piperazine derivatives is described (Elliott, 2011). The crystal structures of some related compounds, viz., 2-[(4-methylpiperazin-1-yl)iminomethyl]phenol (Guo, 2007), 1,4-bis{3-[4-(dimethylamino)benzylideneamino] propyl}piperazine (Xu *et al.*, 2009), 2-methoxy-4-[(4-methylpiperazin-1-yl)-iminomethyl]phenol (Zhou *et al.*, 2011) and 2,4-dibromo-6-[(4-methylpiperazin-1-yl) iminomethyl]phenol (Guo & Qiu, 2007) have been reported. The crystal structures of similar Schiff bases, viz, (1H-indol-3-yl-methylene)- (4-methyl-piperazin-1-yl)-amine (Kavitha *et al.*, 2013*a*) and (4-methyl-piperazin-1-yl)-(2-nitro-benzylidene)-amine (Kavitha *et al.*, 2013*b*) have been reported. The title compound is a Schiff base prepared by the reaction of 1-amino-4-methylpiperazine and 2,3,4-trimethoxy benzaldehyde. In view of the above importance of N-piperazinyl Schiff bases, the title compound, (I), C_15_H_23_N_3_O_3_, has been synthesized and the crystal structure is reported.

The title compound, (I), crystallizes with one independent molecule in the asymmetric unit. In the molecule, the piperazine ring is in a slightly disordered chair conformation (puckering parameters Q, θ, and φ = 0.5691 (15)Å, 176.10 (14)° and 160 (2)°, respectively; (Cremer & Pople, 1975) and is twisted from the mean plane of the phenyl ring with a N2/N3/C5/C6 torsion angle of 177.3 (7)° (Fig. 1). The 4-methoxy substituent with a C10/C9/O3/C15 torsion angle of 5.4 (1)° is almost planar with respect to the mean plane of the phenyl ring while the methoxy groups at positions 2 and 3, with torsion angles of 122.6 (4)° (C6/C7/O1/C13) and -66.1 (4)° (C9/C8/O2/C14), are twisted away from the mean plane of the phenyl ring in anti-clinical and -syn-clinical conformations, respectively. Bond lengths are in normal ranges (Allen *et al.*, 1987). No classical hydrogen bonds or any weak intermolecular interactions are observed.

### 2. Experimental

To a solution of 2,3,4-trimethoxy benzaldehyde (0.98 g, 0.005 mol) in 5 ml of methanol an equimolar amount of (1-amino-4-methyl)piperazine (0.57 g, 0.005 mol) is added dropwise with constant stirring. The mixture was refluxed for eight hours . The solution was evaporated at room temperature to obtain the solid. The solid was then recrystallized using ethylacetate and the crystals were used as such for x-ray diffraction studies (m.p.: 365-369 K).

### 3. Refinement

All of the H atoms were placed in their calculated positions and then refined using the riding model with Atom—H lengths of 0.93Å (CH), 0.97Å (CH_2_) OR 0.96Å (CH_3_). Isotropic displacement parameters for these atoms were set to 1.2 (CH, CH_2_) or 1.5 (CH_3_) times *U*_eq_ of the parent atom. Idealised Me refined as rotating groups.

### Crystal data

**Table:**

C_15_H_23_N_3_O_3_	*D*_x_ = 1.264 Mg m^−^^3^
*M**_r_* = 293.36	Cu *K*α radiation, λ = 1.54184 Å
Orthorhombic, *P**b**c**a*	Cell parameters from 8346 reflections
*a* = 7.84207 (14) Å	θ = 4.5–71.5°
*b* = 14.2305 (3) Å	µ = 0.73 mm^−^^1^
*c* = 27.6218 (5) Å	*T* = 173 K
*V* = 3082.49 (10) Å^3^	Irregular, colourless
*Z* = 8	0.30 × 0.26 × 0.18 mm
*F*(000) = 1264	

### Data collection

**Table:**

Agilent Xcalibur (Eos, Gemini) diffractometer	2978 independent reflections
Radiation source: Enhance (Cu) X-ray Source	2643 reflections with *I* > 2σ(*I*)
Detector resolution: 16.0416 pixels mm^-1^	*R*_int_ = 0.051
ω scans	θ_max_ = 71.3°, θ_min_ = 3.2°
Absorption correction: multi-scan (*CrysAlis PRO* and *CrysAlis RED*; Agilent, 2012)	*h* = −9→9
*T*_min_ = 0.290, *T*_max_ = 1.000	*k* = −14→17
19693 measured reflections	*l* = −33→33

### Refinement

**Table:**

Refinement on *F*^2^	Hydrogen site location: inferred from neighbouring sites
Least-squares matrix: full	H-atom parameters constrained
*R*[*F*^2^ > 2σ(*F*^2^)] = 0.040	*w* = 1/[σ^2^(*F*_o_^2^) + (0.0679*P*)^2^ + 0.5881*P*] where *P* = (*F*_o_^2^ + 2*F*_c_^2^)/3
*wR*(*F*^2^) = 0.113	(Δ/σ)_max_ = 0.001
*S* = 1.04	Δρ_max_ = 0.23 e Å^−^^3^
2978 reflections	Δρ_min_ = −0.17 e Å^−^^3^
195 parameters	Extinction correction: *SHELXL2012* (Sheldrick, 2008), Fc^*^=kFc[1+0.001xFc^2^λ^3^/sin(2θ)]^-1/4^
0 restraints	Extinction coefficient: 0.00092 (14)
Primary atom site location: structure-invariant direct methods	

### Special details

**Table:**

Geometry. All esds (except the esd in the dihedral angle between two l.s. planes) are estimated using the full covariance matrix. The cell esds are taken into account individually in the estimation of esds in distances, angles and torsion angles; correlations between esds in cell parameters are only used when they are defined by crystal symmetry. An approximate (isotropic) treatment of cell esds is used for estimating esds involving l.s. planes.

### Fractional atomic coordinates and isotropic or equivalent isotropic displacement parameters (Å^2^)

**Table:**

	*x*	*y*	*z*	*U*_iso_*/*U*_eq_	
O1	0.29270 (12)	0.40317 (7)	0.53704 (3)	0.0368 (2)	
O2	0.27459 (11)	0.21257 (6)	0.51816 (3)	0.0335 (2)	
O3	0.44344 (12)	0.08844 (6)	0.57497 (3)	0.0352 (2)	
N1	0.44972 (15)	0.77433 (8)	0.70944 (4)	0.0362 (3)	
N2	0.54792 (13)	0.60211 (7)	0.66430 (4)	0.0295 (2)	
N3	0.54620 (13)	0.50677 (7)	0.65387 (4)	0.0302 (2)	
C1	0.44272 (17)	0.76304 (9)	0.65706 (5)	0.0357 (3)	
H1A	0.5484	0.7854	0.6429	0.043*	
H1B	0.3503	0.8008	0.6442	0.043*	
C2	0.41546 (17)	0.66106 (9)	0.64310 (5)	0.0353 (3)	
H2A	0.3045	0.6404	0.6544	0.042*	
H2B	0.4179	0.6550	0.6081	0.042*	
C3	0.56104 (18)	0.61506 (9)	0.71668 (5)	0.0351 (3)	
H3A	0.6555	0.5782	0.7291	0.042*	
H3B	0.4573	0.5931	0.7321	0.042*	
C4	0.58872 (19)	0.71782 (10)	0.72874 (5)	0.0372 (3)	
H4A	0.5946	0.7257	0.7636	0.045*	
H4B	0.6960	0.7388	0.7150	0.045*	
C5	0.46406 (15)	0.47565 (9)	0.61694 (4)	0.0286 (3)	
H5	0.4013	0.5165	0.5976	0.034*	
C6	0.47012 (14)	0.37502 (9)	0.60550 (4)	0.0279 (3)	
C7	0.38193 (14)	0.34008 (9)	0.56495 (4)	0.0276 (3)	
C8	0.37692 (14)	0.24401 (9)	0.55517 (4)	0.0278 (3)	
C9	0.46380 (15)	0.18119 (9)	0.58557 (4)	0.0285 (3)	
C10	0.55834 (16)	0.21556 (9)	0.62441 (4)	0.0318 (3)	
H10	0.6207	0.1746	0.6438	0.038*	
C11	0.55890 (16)	0.31101 (9)	0.63400 (4)	0.0309 (3)	
H11	0.6207	0.3331	0.6604	0.037*	
C12	0.4689 (2)	0.87309 (11)	0.72222 (6)	0.0494 (4)	
H12A	0.5711	0.8974	0.7078	0.074*	
H12B	0.4757	0.8792	0.7568	0.074*	
H12C	0.3724	0.9078	0.7105	0.074*	
C13	0.3389 (2)	0.40626 (11)	0.48695 (5)	0.0441 (3)	
H13A	0.2757	0.3594	0.4695	0.066*	
H13B	0.4588	0.3941	0.4837	0.066*	
H13C	0.3132	0.4673	0.4741	0.066*	
C14	0.36197 (19)	0.16466 (11)	0.47953 (5)	0.0398 (3)	
H14A	0.2870	0.1586	0.4522	0.060*	
H14B	0.3962	0.1034	0.4903	0.060*	
H14C	0.4610	0.2001	0.4703	0.060*	
C15	0.51186 (19)	0.02173 (9)	0.60812 (5)	0.0400 (3)	
H15A	0.4813	−0.0405	0.5980	0.060*	
H15B	0.4666	0.0333	0.6399	0.060*	
H15C	0.6338	0.0274	0.6089	0.060*	

### Atomic displacement parameters (Å^2^)

**Table:**

	*U*^11^	*U*^22^	*U*^33^	*U*^12^	*U*^13^	*U*^23^
O1	0.0377 (5)	0.0364 (5)	0.0363 (5)	0.0096 (4)	−0.0095 (4)	−0.0018 (4)
O2	0.0263 (4)	0.0382 (5)	0.0360 (5)	0.0000 (3)	−0.0049 (3)	−0.0069 (4)
O3	0.0389 (5)	0.0280 (5)	0.0388 (5)	−0.0006 (4)	−0.0020 (4)	0.0006 (4)
N1	0.0396 (6)	0.0337 (6)	0.0353 (6)	−0.0016 (4)	0.0053 (4)	−0.0043 (5)
N2	0.0288 (5)	0.0294 (5)	0.0303 (5)	−0.0002 (4)	−0.0043 (4)	−0.0007 (4)
N3	0.0277 (5)	0.0297 (5)	0.0331 (5)	−0.0011 (4)	−0.0009 (4)	−0.0004 (4)
C1	0.0374 (7)	0.0333 (7)	0.0366 (7)	0.0029 (5)	−0.0056 (5)	−0.0002 (5)
C2	0.0347 (6)	0.0356 (7)	0.0355 (6)	0.0017 (5)	−0.0098 (5)	−0.0020 (5)
C3	0.0395 (7)	0.0360 (7)	0.0298 (6)	−0.0035 (5)	−0.0063 (5)	0.0019 (5)
C4	0.0438 (7)	0.0399 (7)	0.0278 (6)	−0.0075 (6)	−0.0038 (5)	−0.0022 (5)
C5	0.0250 (6)	0.0323 (6)	0.0286 (6)	0.0000 (5)	0.0003 (4)	0.0007 (5)
C6	0.0234 (5)	0.0328 (6)	0.0276 (6)	−0.0008 (4)	0.0031 (4)	−0.0001 (5)
C7	0.0220 (5)	0.0323 (6)	0.0285 (6)	0.0021 (4)	0.0015 (4)	0.0016 (5)
C8	0.0213 (5)	0.0352 (7)	0.0269 (6)	−0.0012 (4)	0.0015 (4)	−0.0022 (5)
C9	0.0260 (6)	0.0293 (6)	0.0300 (6)	−0.0006 (4)	0.0045 (4)	−0.0010 (5)
C10	0.0322 (6)	0.0335 (7)	0.0297 (6)	0.0031 (5)	−0.0026 (5)	0.0040 (5)
C11	0.0297 (6)	0.0363 (7)	0.0267 (6)	−0.0005 (5)	−0.0022 (4)	−0.0017 (5)
C12	0.0702 (11)	0.0380 (8)	0.0400 (8)	−0.0030 (7)	0.0069 (7)	−0.0063 (6)
C13	0.0510 (8)	0.0443 (8)	0.0370 (7)	0.0011 (6)	−0.0104 (6)	0.0083 (6)
C14	0.0409 (7)	0.0442 (8)	0.0343 (7)	0.0017 (6)	−0.0051 (5)	−0.0104 (6)
C15	0.0428 (7)	0.0310 (7)	0.0462 (8)	0.0045 (6)	−0.0006 (6)	0.0029 (6)

### Geometric parameters (Å, º)

**Table:**

O1—C7	1.3747 (15)	C5—H5	0.9300
O1—C13	1.4308 (17)	C5—C6	1.4673 (17)
O2—C8	1.3745 (14)	C6—C7	1.4071 (17)
O2—C14	1.4397 (16)	C6—C11	1.3908 (18)
O3—C9	1.3614 (15)	C7—C8	1.3941 (18)
O3—C15	1.4239 (16)	C8—C9	1.4032 (18)
N1—C1	1.4570 (17)	C9—C10	1.3928 (18)
N1—C4	1.4556 (19)	C10—H10	0.9300
N1—C12	1.4567 (18)	C10—C11	1.3838 (18)
N2—N3	1.3871 (14)	C11—H11	0.9300
N2—C2	1.4579 (16)	C12—H12A	0.9600
N2—C3	1.4623 (16)	C12—H12B	0.9600
N3—C5	1.2852 (16)	C12—H12C	0.9600
C1—H1A	0.9700	C13—H13A	0.9600
C1—H1B	0.9700	C13—H13B	0.9600
C1—C2	1.5167 (18)	C13—H13C	0.9600
C2—H2A	0.9700	C14—H14A	0.9600
C2—H2B	0.9700	C14—H14B	0.9600
C3—H3A	0.9700	C14—H14C	0.9600
C3—H3B	0.9700	C15—H15A	0.9600
C3—C4	1.5154 (18)	C15—H15B	0.9600
C4—H4A	0.9700	C15—H15C	0.9600
C4—H4B	0.9700		
			
C7—O1—C13	115.69 (10)	O1—C7—C6	117.76 (11)
C8—O2—C14	115.31 (10)	O1—C7—C8	121.16 (11)
C9—O3—C15	117.64 (10)	C8—C7—C6	120.97 (11)
C4—N1—C1	109.32 (10)	O2—C8—C7	118.68 (11)
C4—N1—C12	111.54 (12)	O2—C8—C9	121.42 (11)
C12—N1—C1	110.54 (11)	C7—C8—C9	119.68 (11)
N3—N2—C2	118.20 (10)	O3—C9—C8	115.60 (11)
N3—N2—C3	109.23 (10)	O3—C9—C10	124.65 (11)
C2—N2—C3	112.02 (10)	C10—C9—C8	119.72 (11)
C5—N3—N2	120.44 (11)	C9—C10—H10	120.2
N1—C1—H1A	109.4	C11—C10—C9	119.59 (11)
N1—C1—H1B	109.4	C11—C10—H10	120.2
N1—C1—C2	111.30 (11)	C6—C11—H11	118.9
H1A—C1—H1B	108.0	C10—C11—C6	122.20 (11)
C2—C1—H1A	109.4	C10—C11—H11	118.9
C2—C1—H1B	109.4	N1—C12—H12A	109.5
N2—C2—C1	110.37 (10)	N1—C12—H12B	109.5
N2—C2—H2A	109.6	N1—C12—H12C	109.5
N2—C2—H2B	109.6	H12A—C12—H12B	109.5
C1—C2—H2A	109.6	H12A—C12—H12C	109.5
C1—C2—H2B	109.6	H12B—C12—H12C	109.5
H2A—C2—H2B	108.1	O1—C13—H13A	109.5
N2—C3—H3A	109.6	O1—C13—H13B	109.5
N2—C3—H3B	109.6	O1—C13—H13C	109.5
N2—C3—C4	110.43 (10)	H13A—C13—H13B	109.5
H3A—C3—H3B	108.1	H13A—C13—H13C	109.5
C4—C3—H3A	109.6	H13B—C13—H13C	109.5
C4—C3—H3B	109.6	O2—C14—H14A	109.5
N1—C4—C3	110.21 (11)	O2—C14—H14B	109.5
N1—C4—H4A	109.6	O2—C14—H14C	109.5
N1—C4—H4B	109.6	H14A—C14—H14B	109.5
C3—C4—H4A	109.6	H14A—C14—H14C	109.5
C3—C4—H4B	109.6	H14B—C14—H14C	109.5
H4A—C4—H4B	108.1	O3—C15—H15A	109.5
N3—C5—H5	120.3	O3—C15—H15B	109.5
N3—C5—C6	119.41 (11)	O3—C15—H15C	109.5
C6—C5—H5	120.3	H15A—C15—H15B	109.5
C7—C6—C5	120.03 (11)	H15A—C15—H15C	109.5
C11—C6—C5	122.24 (11)	H15B—C15—H15C	109.5
C11—C6—C7	117.73 (11)		
			
O1—C7—C8—O2	−2.82 (16)	C5—C6—C7—C8	−175.85 (10)
O1—C7—C8—C9	−177.37 (10)	C5—C6—C11—C10	177.07 (11)
O2—C8—C9—O3	1.80 (16)	C6—C7—C8—O2	173.26 (10)
O2—C8—C9—C10	−176.27 (11)	C6—C7—C8—C9	−1.30 (16)
O3—C9—C10—C11	−174.78 (11)	C7—C6—C11—C10	−1.90 (18)
N1—C1—C2—N2	−55.98 (15)	C7—C8—C9—O3	176.20 (10)
N2—N3—C5—C6	177.42 (10)	C7—C8—C9—C10	−1.88 (17)
N2—C3—C4—N1	58.01 (14)	C8—C9—C10—C11	3.11 (18)
N3—N2—C2—C1	−177.82 (10)	C9—C10—C11—C6	−1.20 (19)
N3—N2—C3—C4	171.89 (10)	C11—C6—C7—O1	179.34 (10)
N3—C5—C6—C7	−179.49 (11)	C11—C6—C7—C8	3.14 (17)
N3—C5—C6—C11	1.57 (18)	C12—N1—C1—C2	−177.65 (12)
C1—N1—C4—C3	−59.88 (14)	C12—N1—C4—C3	177.56 (11)
C2—N2—N3—C5	19.32 (17)	C13—O1—C7—C6	122.85 (12)
C2—N2—C3—C4	−55.19 (14)	C13—O1—C7—C8	−60.96 (15)
C3—N2—N3—C5	148.93 (11)	C14—O2—C8—C7	119.41 (12)
C3—N2—C2—C1	53.86 (14)	C14—O2—C8—C9	−66.14 (15)
C4—N1—C1—C2	59.20 (14)	C15—O3—C9—C8	−172.57 (11)
C5—C6—C7—O1	0.35 (16)	C15—O3—C9—C10	5.40 (17)
